# Benchmarking the Human Leukocyte Antigen Typing Performance of Three Assays and Seven Next-Generation Sequencing-Based Algorithms

**DOI:** 10.3389/fimmu.2021.652258

**Published:** 2021-03-31

**Authors:** Ping Liu, Minya Yao, Yu Gong, Yunjie Song, Yanan Chen, Yizhou Ye, Xiao Liu, Fugen Li, Hua Dong, Rui Meng, Hao Chen, Aiwen Zheng

**Affiliations:** ^1^ Department of Oncology, The Second Xiangya Hospital of Central South University, Changsha, China; ^2^ The First Affiliated Hospital of Zhejiang University, School of Medicine, Hangzhou, China; ^3^ Department of Urology, Second Affiliated Hospital, Zhejiang University College of Medicine, Hangzhou, China; ^4^ The Bioinformatics Department, 3DMed Inc., Shanghai, China; ^5^ Tongji Medical College, Huazhong University of Science and Technology, Wuhan, China; ^6^ Cancer Hospital of the University of Chinese Academy of Sciences (Zhejiang Cancer Hospital), Hangzhou, China; ^7^ Institute of Cancer and Basic Medicine (IBMC), Chinese Academy of Sciences, Hangzhou, China

**Keywords:** human leukocyte antigen, accuracy, next-generation sequencing, algorithms, benchmark

## Abstract

With the great progress made recently in next generation sequencing (NGS) technology, sequencing accuracy and throughput have increased, while the cost for data has decreased. Various human leukocyte antigen (HLA) typing algorithms and assays have been developed and have begun to be used in clinical practice. In this study, we compared the HLA typing performance of three HLA assays and seven NGS-based HLA algorithms and assessed the impact of sequencing depth and length on HLA typing accuracy based on 24 benchmarked samples. The algorithms HISAT-genotype and HLA-HD showed the highest accuracy at both the first field and the second field resolution, followed by HLAscan. Our internal capture-based HLA assay showed comparable performance with whole exome sequencing (WES). We found that the minimal depth was 100X for HISAT-genotype and HLA-HD to obtain more than 90% accuracy at the third field level. The top three algorithms were quite robust to the change of read length. Thus, we recommend using HISAT-genotype and HLA-HD for NGS-based HLA genotyping because of their higher accuracy and robustness to read length. We propose that a minimal sequence depth for obtaining more than 90% HLA typing accuracy at the third field level is 100X. Besides, targeting capture-based NGS HLA typing may be more suitable than WES in clinical practice due to its lower sequencing cost and higher HLA sequencing depth.

## Introduction

The human leukocyte antigen (HLA), commonly referred as major histocompatibility complex (MHC) which is often found in all jawed vertebrates (1), is located within a region of approximately 4 M in length on the short arm of human chromosome 6 (6p21.3), with more than 200 protein-coding genes (2). Except for identical twins, no two individuals have exactly the same HLA. Therefore, HLA is also known as the “identity card” of the human cell. It is a marker for the mutual recognition of immune cells in different individuals. HLA gene products are expressed on different cell surfaces and play a key role in antigen presentation and immune signaling. HLA mainly includes three regions, namely HLA class I, HLA class II, and HLA class III. HLA class I genes include HLA-A, HLA-B, and HLA-C, which are distributed on almost all nucleated cell surfaces with the highest lymphocyte surface density (3). HLA class II genes include the HLA-D family, mainly HLA-DP, HLA-DQ, and HLA-DR, which are mainly distributed on the surface of professional antigen-presenting cells such as B lymphocytes, macrophages, and dendritic cells. The HLA class III gene contains approximately 75 genes, most of which are of unknown function. HLA class I and HLA class II genes are molecules that encode binding and presenting antigens, allowing cytotoxic T lymphocytes to bind to mature HLA cell surface proteins *via* antigen-binding channels. HLA class I genes mainly encode antigens to CD8+ T cells, and HLA class II genes mainly encode antigens to CD4+ T cells.

HLA has been widely used in hematopoietic stem cell transplantation (HSCT), detection of susceptibility genes in immune-related diseases, and drug allergy testing. HSCT was treated as the last resort therapeutic approach for a wide range of malignant and non-malignant diseases and suitable donor selection is determined with the utilization of HLA typing and highly similar HLA alleles improve the clinical outcome and reduce the risk of rejection (4). According to USA standards, 8/8 match for the loci HLA-A, HLA-B, HLA-C, and HLA-DRB1 is necessary for a allele-matched donor selection, and single mismatch for these regions are associated with 25% increase in post-transplant complications (5). But in most European centers the gold standard is to look for 10/10 match for HLA-A, HLA-B, HLA-C, HLA-DRB1, and HLA-DQB1 (6). The definition of “HLA matching” depends on the HLA typing resolution, mainly include: Low resolution typing or first field typing, which is equivalent to serological typing and refers to a group of alleles (alleles family); High resolution typing, or second field typing, which refers to one or a set of alleles for the same antigen binding site; Allele level typing, or all field typing, which refers to the exact nucleotide sequence of a HLA gene; Other levels of resolution, which refers to intermediate level of typing and could define specific subtypes. Currently, high resolution typing of HLA genes were recommended by National Marrow Donors Program (NMDP) (5). Thus, HLA typing at the high resolution level is of great clinical significance.

Recent studies have demonstrated that HLA typing complexity is associated with the efficacy of cancer immune checkpoint blockade (ICB) (7). Furthermore, the combined effect of HLA class I heterozygosity and tumor mutation burden (TMB) on improved survival is greater as compared with mutation load alone (7, 8). Researchers have also sequenced the CDR3 of the hypervariable region of the T cell receptor (TCR) and found that the TCR CDR3’s tumor-associated clones are significantly elevated in patients with greater heterogeneity of the HLA class of molecular sites (7). That is to say, in the treatment of ICB, the diversity of HLA molecules in patients will affect the clonal expansion of T cells against new tumor antigens and thus affect the therapeutic effect (9). The highly polymorphic HLA genes present unique challenges for the development of molecular approaches to genotype HLA alleles. According to the traditional method, both alleles of a particular HLA locus are PCR amplified and Sanger-sequenced together, resulting in multiple heterozygous positions in the electropherogram tracing. With the development of next-generation sequencing (NGS) technology, each fragment of HLA DNA is amplified and sequenced independently, dramatically reducing the phase ambiguities encountered with Sanger sequencing. Since 2009, many different approaches for HLA genotyping by the NGS method have been reported using a variety of capture strategies and sequencing platforms (10–15). While whole exome sequencing is the gold standard in some case, such as measurement of TMB in clinic, targeted next-generation sequencing panels might be ideal for HLA typing which allows us to customize probes that only include genomic regions of HLA genes, and sequence HLA gene at a much higher depth but lower input amounts than WES. Many bioinformatics approaches have also been developed to produce HLA genotyping information from amplicon-based NGS, targeted capture (e.g., whole-exome sequencing) and non-targeted whole-genome sequencing (16–23) (software used in this study are listed in **Table 1**). All these algorithms can be generally divided into two categories: alignment-based methods and assembly-based methods. The former category aligns the sequencing data to the HLA reference database IPD-IMGT/HLA (24, 25) and predicts HLA genotypes using probabilistic models (26), whereas the latter assembles reads into contigs and aligns those to the known HLA allele reference sequences. Several studies have been conducted to compare the accuracy of different software (26–30). Bauer et al. evaluated the HLA typing accuracy of five computational methods on three different data sets, finding that PHLAT has the highest accuracy, and sequencing coverage has a weak correlation with accuracy (26). However, no conclusions have been made regarding several critical questions: Which HLA typing assay is more suitable in a clinical context? Whether HLA typing algorithms were biased towards a specific NGS assay? What are the basic sequencing requirements for accurate HLA genotyping? To answer these questions, we evaluated the performance of different combinations of HLA NGS typing assays and software using our in-house benchmarking dataset.

**Table 1:** HLA-typing software used in this study.

Software	Resolution	Programming	Year	Journal	Cited
HLAminer	4	Perl	2012	Genome Medicine	83
seq2HLA	4	Python, R	2012	Genome Medicine	93
HLAforest	8	Perl	2013	PLOS ONE	28
HLA-VBSeq	8	Java	2015	BMC Genomics	36
HLA-HD	6	Shell	2017	Human mutation	15
HLAscan	8	Python	2017	BMC Bioinformatics	22
HISAT-genotype	8	C++, Python	2019	Nature Biotechnology	81

## Materials And Methods

### Sample Preparation

A total of 24 samples were collected, and genomic DNA was extracted from white blood cell samples using a QIAamp DNA Blood Mini Kit (QIAGEN, Cat. No. 51106). DNA fragments of approximately 200 bp were selected from sheared genomic DNA for library preparation and sequencing. Another 998 Chinese patient samples were collected from Apr. 3, 2018, to Jan. 27, 2019, for HLA typing by an internally developed HLA assay.

### HLA Genotyping Assays

HLA genotyping from the amplicon assay NGSgo-AmpX was used as the benchmark reference. NGSgo-AmpX consists of dedicated primer sets for the amplification of individual HLA genes, enabling the amplification of the following HLA genes: Class I: HLA-A, HLA-B, and HLAC-C; and Class II: HLA-DRB1 and HLA-DQB1 (GenDx, Utrecht, Netherlands). Three capture-based assays include 1) Agilent SureSelect Human All Exon V5+UTR kits and paired-end sequencing (150PE) strategies were carried out using standard Illumina protocols on an Illumina HiSeq X10 system (WES for short). Each sample met the average depth over 100X and capture on-target ratio >50%. 2) IDT xGen^®^ Exome Research Panel kits and paired-end sequencing (150PE) strategies were carried out using standard Illumina protocols on an Illumina HiSeq X10 system (Bofuri for short). Each sample met the average depth over 100X and capture on-target ratio >60% (10 samples were not available). 3) 3DMed Inc. in-house designed and developed HLA specific probes and paired-end sequencing (150PE) was carried out using standard Illumina protocols on an Illumina HiSeq X10 system (Internal for short). Each sample met the average depth over 100X and capture on-target ratio >60%. The raw fastq files from Miseq sequencing were subsequently processed and validated by the vendor independently, and used as the benchmarked result for HLA typing.

### NGS-Based HLA Genotyping Algorithms

We compared seven publicly available algorithms for HLA typing: seq2HLA (16), HLAminer (17), HLAscan (20), HLA-VBSeq (21), HLA-HD (22), HLAforest (30), and HISAT-genotype (31). The algorithms were chosen considering their accessibility and number of citations. For HLAscan, raw sequence data were first mapped to the human reference genome UCSC hg19, and reads from chr6 of the BAM files were then generated as an input, the database file was directly downloaded along with the program from github, and other parameters were set to default; for HLA-VBSeq, HLA v2 database and the same instruction on the website were used for HLA typing (http://nagasakilab.csml.org/hla/); for HISAT-genotype, we used raw sequence files as an input, and two program “hisatgenotype_extract_reads.py” and “hisatgenotype.py” was used to HLA typing; for HLAminer, seq2HLA, HLA-HD and HLAforest, raw fastq file was used as input, and all these algorithms were run with default parameters; HLA typing accuracy was defined as the percentage of correctly identified alleles among all the reference alleles. We tested the HLA typing accuracy of all seven algorithms and selected the top three with the highest overall accuracy for our read depth and length evaluation.

### Linux Server Hardware Configuration

All software were run on a Linux server (CentOS6.5, kernel version: 2.6.32-431.11.2) with the hardware configuration as follows: Intel(R) Xeon(R) CPU E5-2650 v3 @ 2.30 GHz/250 GB RAM/more than 10 TB disk space. R software was used for statistical analysis and plot creation (version: 3.6.1).

## Results

### HLA Typing Workflow

**Figure 1:**
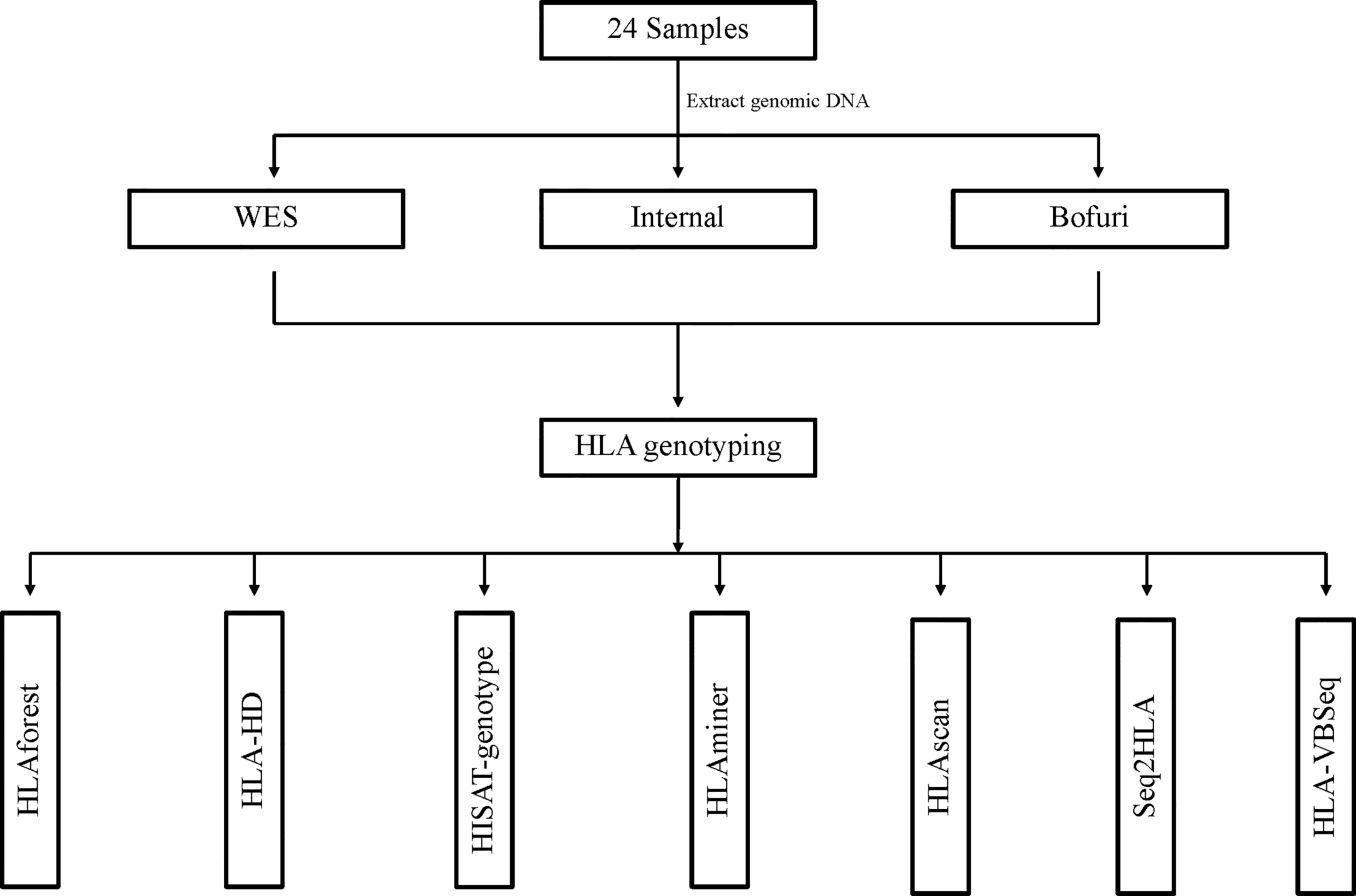
Workflow of HLA typing using benchmarked data sets. All HLA typing algorithms were run with default parameters.

Our HLA typing workflow is outlined in **Figure 1**, including DNA isolation, library preparation, high-throughput sequencing, and bioinformatics analysis. Three HLA typing NGS assays—whole-exome sequence (WES), IDT xGen^®^ Exome Research Panel (Bofuri), and 3DMed internal panel (Internal)—were selected to generate benchmarked HLA sequencing libraries. Genomic DNA of 24 samples was collected, and then libraries were prepared and sequenced using PE150bp on an Illumina HiSeq X10 system. For the NGS-based HLA genotyping, each sample was determined by seven software, namely seq2HLA, HLAminer, HLAscan, HLA-VBSeq, HLA-HD, HLAforest, and HISAT-genotype, and default parameters were used for all software. Benchmarking HLA results of the 24 samples (**Supplementary Table 1**) were produced by amplicon assay NGSgo-AmpX plus Miseq sequencing.

### HLA Typing Accuracy for All Assay-Software Combinations

**Figure 2:**
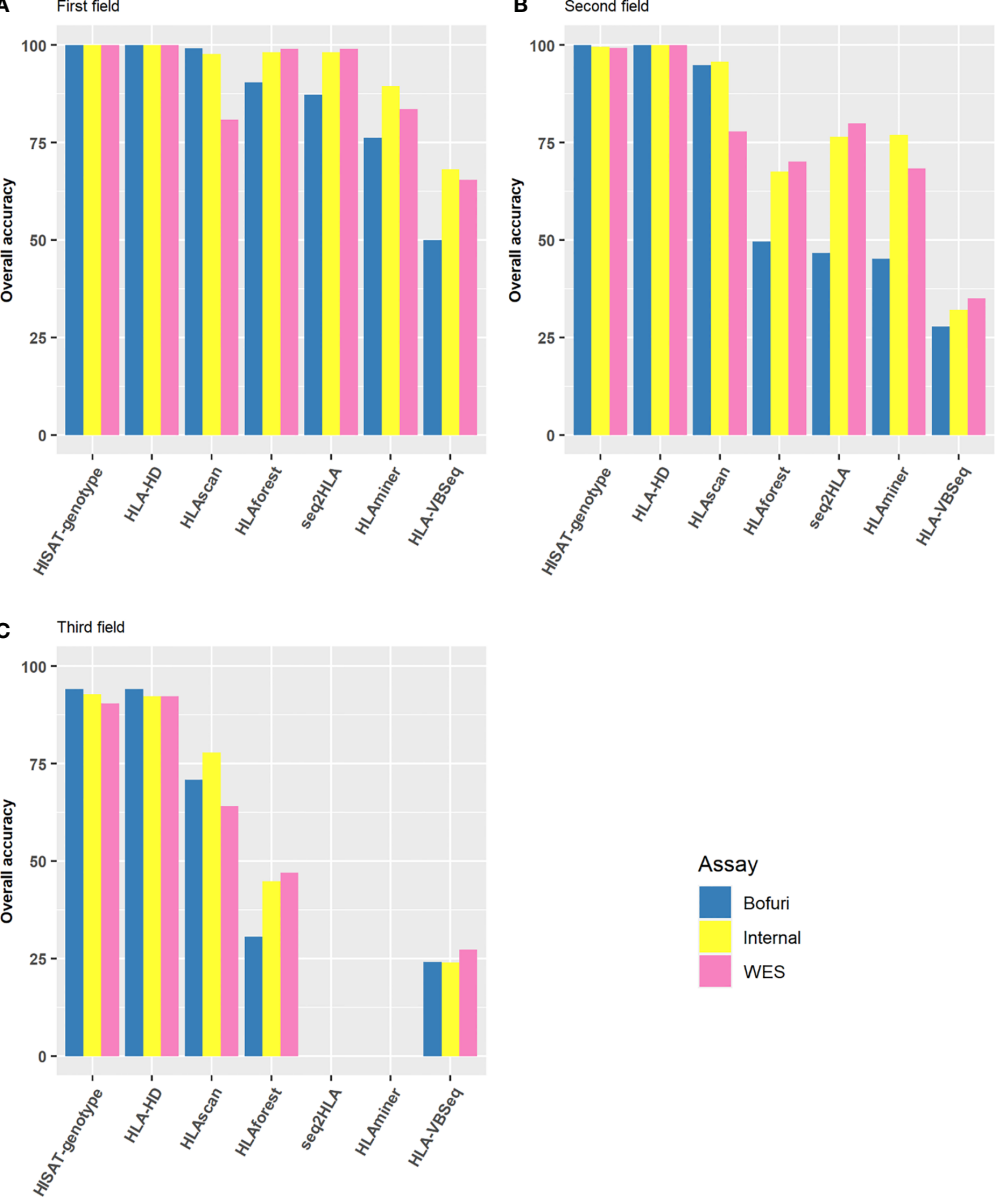
Performance of HLA typing algorithms and the three different HLA assays. Accuracy of HLA alleles typed at **(A)** the first field level; **(B)** the second field level; **(C)** the third field level based on the seven algorithms and three capture assays. Accuracy was calculated by the fraction of total number alleles that were correctly typed.

As a preliminary screening, we first compared the HLA typing accuracy of all possible assay-software combinations at the first, second, and third field levels. The results were much more discordant among different algorithms than among the capture assays used. At the first field level, six of the seven algorithms had an overall accuracy of higher than 75% no matter which assay was used (**Figure 2A**). HLA-HD and HISAT-genotype had almost perfect accuracy, whereas the accuracy of HLAVBseq was lower (the accuracy was 68, 65, and 50% for Internal, WES, and Bofuri, respectively). As the HLA resolution increased from the first field to the second field levels, the accuracy of HLA tying gradually decreased (**Figures 2B, C**; HLA typing results for HLAminer and HLAseq2HLA at the third field level were not available). Only HLA-HD and HISAT-genotype showed greater than 75% accuracy at the third field level. Among all three assays used, the overall accuracy of Bofuri was the lowest, and our internal NGS assays showed comparable performance compared with WES when other algorithms than HISAT-genotype and HLA-HD were used. On the other hand, HISAT-genotype and HLA-HD are quite robust to the assays and resolutions. Above all, our research showed that the choice of the HLA typing algorithms contribute most to the accuracy and target-captured panel could match performance of WES.

### Computer Resource Consumption

**Figure 3:**
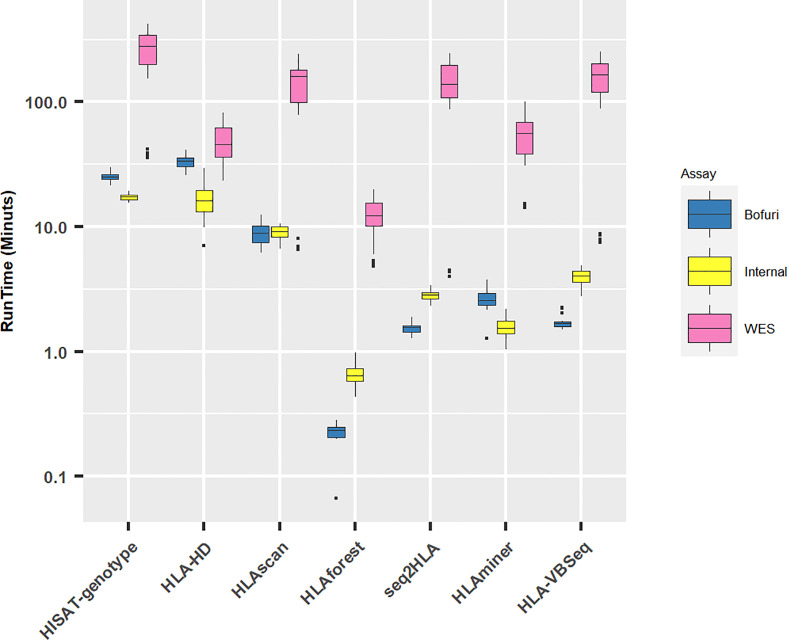
Running time for different HLA typing software. Y axis is plotted in log10 scale.

All HLA programs were run on a Linux server with eight threads if possible. As expected, with the increase in panel sizes of NGS capture assays, the running time for all the software increased (**Figure 3**). Unsurprisingly, the running time for WES increased exponentially compared with the other two assays (median running time: WES, 77 min; Internal, 4 min; Bofuri, 3 min). For the other two assays, the most time-consuming algorithms were HLA-HD and HISAT-genotype.

### Discordant HLA Typing Patterns Across Algorithms

**Figure 4:**
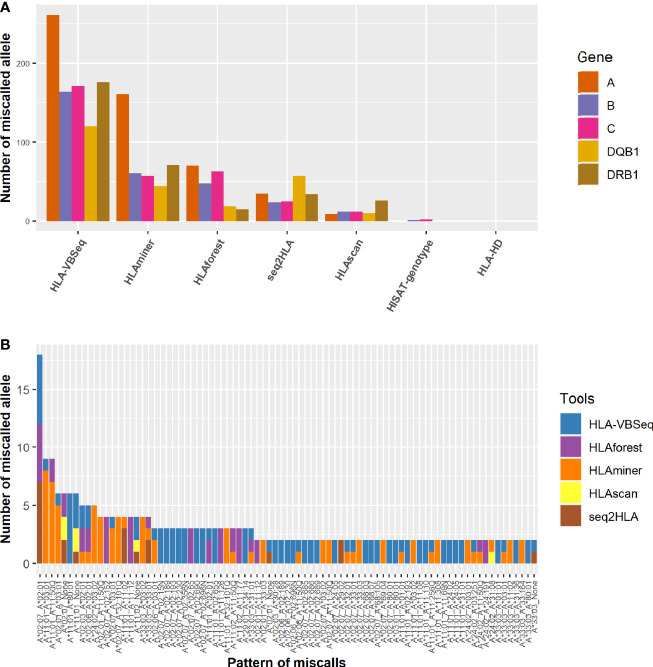
Distribution of the pattern of genotyping errors in HLA-A genes. **(A)** The number of miscalled alleles by each algorithm grouped by the HLA genes. **(B)** The pattern of discordant HLA-A alleles at the second field level. None, not determined by the algorithms.

We investigated the specific patterns of discordance in each algorithm. Among all the algorithms, HLA-VBSeq had the highest number of miscalled HLA typing at the second field level, followed by HLAminer (**Figure 4A**). Out of the five HLA genes, HLA-A gene was the most frequently miscalled gene, and the most discordant pattern was HLA-A*02:07 to HLA-A*02:01 (**Figure 4B**). Each algorithm had biases on ratios of miscalled HLA typing within specific serological allele groups. For example, 81% (57 out of 70) HLA-A miscalled errors observed in HLAforest were within the same serological allele group, whereas the ratio decreased to less than 15% for HLAscan, HLA-HD, and HISAT-genotypes (**Supplementary Table 2**).

### The Impact of Sequence Depth and Length on HLA Typing Accuracy

Based on the above evaluations, we focused on the three algorithms with the highest accuracies, that is, HISAT-genotype, HLA-HD, and HLAscan, to investigate the impact of read length and read depth on HLA typing.

**Figure 5:**
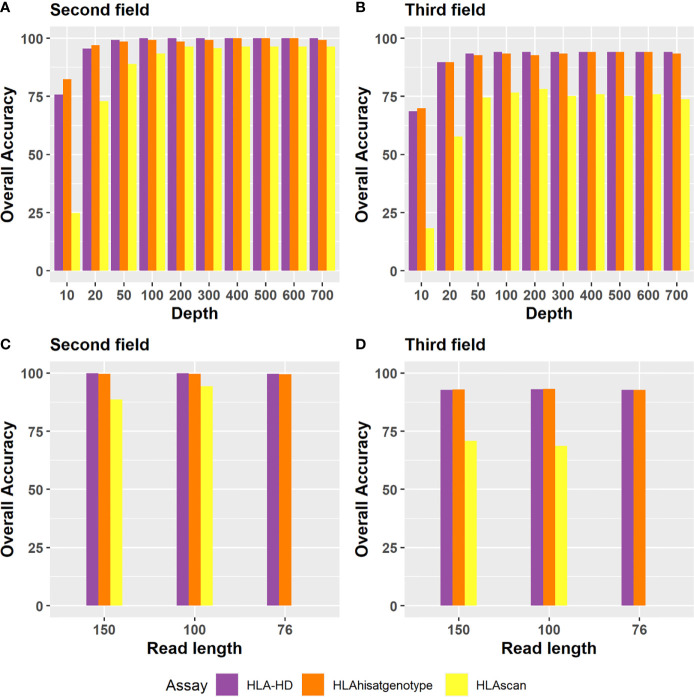
Accuracy of the three tools for HLA typing at the second field or the third field resolution for different depths and read lengths. Depth evaluation at **(A)** the second field level; **(B)** the third field level. For sequence depth evaluation, alignment files of the 24 Bofuri samples were down-sampled from 700X to 10X based on the raw depths of HLA genes. **(C, D)** are the overall HLA typing accuracy at the second field and the third field level, respectively, while the read length decreased from 150 bp to 76 bp.

Regarding the depth evaluation, when the sequencing data of Bofuri were down-sampled from 700X to 10X, the accuracies of HLA-HD and HISAT-genotype at the second field level were still above 95% at 50X, and then they decreased gradually when the sequence depths were less than 50X (**Figure 5A**). The overall accuracy of HLAscan was lower than the other two algorithms. The required sequence depth for HLA-HD and HISAT-genotype to get more than 90% HLA typing accuracy was above 100X at both the second and the third field levels (**Figure 5B**).

Regarding the read length evaluation, we manually generated paired-end 100 bp (PE100) and paired-end 75 bp (PE75) sequence data based on paired-end 150 bp (PE150) using an in-house pipeline which trimmed the sequence from both sides randomly. When the read length decreased from PE150 to PE100 and PE75, the overall HLA typing accuracy was quite similar for each algorithm, except that HLAscan had lower accuracy (**Figures 5C, D**), which demonstrated that the selected three HLA typing algorithms were robust to the read length.

### HLA Typing Performance in Validation Datasets

**Figure 6:**
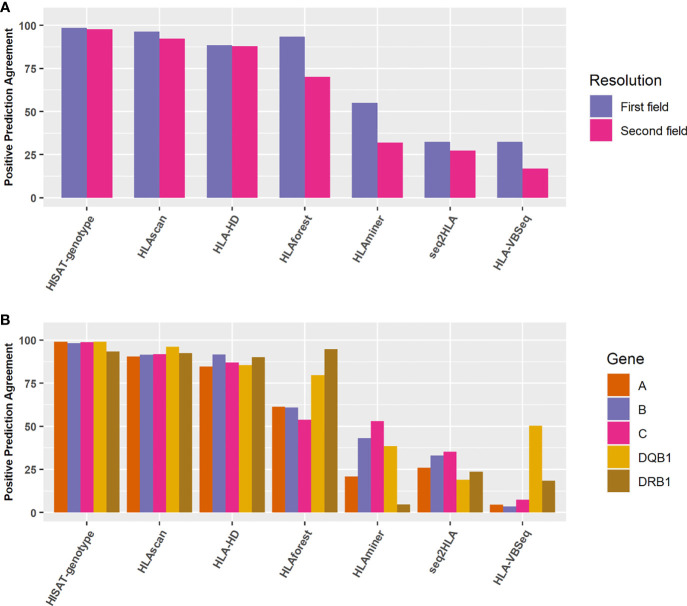
Positive prediction agreement of the seven algorithms for HLA typing at different resolutions and genes. **(A)** Agreement for the seven algorithms at the first field or the second field levels. **(B)** Agreement for the seven algorithms for different HLA genes at the second field level.

We selected another 998 Chinese population samples sequenced by the 3DMed internal developed assay. The reference HLA typing results were defined as the most concordant HLA types called by these seven algorithms. To verify this approach, we compared the most concordant HLA genotypes predicted by different algorithms of the 24 benchmark samples with their reference HLA types at the second field level, and found relatively good consistency between them (The overall accuracy is 0.974, 0.965, 0.970 for internal, WES, and Bofuri respectively), which demonstrated the feasibility of this strategy. For the 998 validation samples, HISAT-genotype, HLA-HD, and HLAscan showed higher accuracy than other algorithms again, and no obvious difference was found for the five HLA genes when HISAT-genotype, HLAscan, and HLA-HD were selected (**Figures 6A, B**), reaffirming our comparison results on HLA typing accuracy.

## Discussion

In this study, we performed a benchmarked analysis of HLA typing based on seven algorithms and three capture-based sequencing methods. As we stressed, the aim of this study is to identify the best technical combination of NGS-based HLA genotyping in clinical context, rather than to evaluate different algorithms. The algorithms which performed not well in this study could be the best choice in a different application context. We found that the choice of NGS-based HLA typing algorithm and the sequencing depth contributed most to the overall HLA typing accuracy. Among the seven algorithms tested, HLA-HD and HISAT-genotype displayed the highest overall accuracies at both the second field and the third field resolutions, which is of great importance in clinic, especially in HSCT where a matched donor was found mainly by HLA typing. When no 10/10 (European standard) or 8/8 (USA standard) matched donor is found, looking for 9/10 or 7/8 matched donor is necessary for HSCT (6). Thus, the NGS based HLA typing accuracy for HSCT should be no less than 90% at the high-resolution level. In this case, HISAT-genotype and HLA-HD may be a good choice. HLA-HD constructed an extensive dictionary of HLA alleles and calculated a score based on weighted read counts to select the most suitable pair of alleles (22). The high accuracy of HLA-HD was more likely related to its elaborate reference database. For HISAT-genotype, it had not only higher HLA typing accuracy but also could be used in CYP (cytochrome P450) typing and V(D)J [variable (V), diversity (D), and joining (J) recombination] typing, which have broad clinical applications. Besides, it could provide fourth field level HLA genotyping, although no reference HLA genotype was available to evaluate the accuracy. A recent study showed that the HLA matched status changed in 29% of pairs after ultra-high resolution (UHR) HLA typing using Pacific Biosciences Single Molecule Real-Time sequencing (Menlo Park, CA, USA), and had significant improved clinic benefit (32), demonstrating that allele level typing (or all field typing) using NGS or other technologies might be an important trend in the field of HSCT. Thus, HISAT-genotype may be a good choice in the HSCT field because of its high accuracy and high resolution.

NGS-based HLA typing can type HLA alleles on each homologous chromosome and can function at higher HLA resolutions, but it is also limited by read length and read depth because of the highly polymorphic nature of the HLA system (26). For example, Ka et al. (20) found that read depth is a critical factor for successful HLA typing by HLAscan and recommended a coverage depth over 90X to ensure 100% predictive accuracy for clinical use, whereas in another accuracy evaluation study of five HLA typing methods, only a weak Pearson correlation between HLA typing accuracy and coverage was found (26). In this study, we evaluated the impact of read depth and read length on the HLA typing accuracy of three algorithms, and the result showed that HLA typing accuracy decreased gradually when the sequence depth was down-sampled from 700X to 10X regardless of which algorithm was used, demonstrating that read depth was a critical factor for accurate HLA typing. To achieve more than 90% HLA typing accuracy at the second field level, the minimal read depth was 50X for the three algorithms used, whereas 100X read depth was needed for HLA-HD and HISAT-genotype to obtain 90% overall accuracy at the third field level.

Though HLA genotyping accuracy was generally concordant among the three NGS assays, our internal capture-based assay showed comparable performance compared with WES, no matter which algorithms were used. Our internal assay designed exon probes of 10 HLA genes (HLA-A, HLA-B, HLA-C, HLA-DPA1, HLA-DPB1, HLA-DQA1, HLA-DQB1, HLA-DRA, HLA-DRB1, and HLA-DRB5). The geographic range is the union of the coding regions of all possible transcripts of the gene. Design rationale included but were not limited to the following (1): For exons longer than the length of the probe, the target area is completely covered by overlapped probes, and the overlaps are larger than 60 nt (2). Each probe was aligned to the whole genome by BLAT (33). The total score was calculated based on the number of hits. The higher the score, the worse the probe specificity. Probes with scores greater than 2 were not considered (3). Probes were not considered in regions of homologous repeats (e.g., SINE, LINE, LTR, etc.). A well-designed probe may improve probe specificity and HLA exon coverage, thus contributing to the accuracy of NGS-based HLA genotyping. Though the accuracy of HLA typing was similar between WES and capture-based assay, capture-based assay is more cost-effective than WES since it only sequence HLA gene. Besides, the sequencing and data analysis speed of capture-based assay is much faster, which shorten the overall turnaround time and more feasible in clinic.

Different algorithms showed different miscall patterns, with HLA-A*02:07 to HLA-A*02:01 being the most widely miscalled allele by HLAforest, seq2HLA, and HLA-VBSeq. It has been reported that the only difference in the peptide sequence between HLA-A*02:01 and HLA-A*02:07 is the 123rd amino acid, which is either Tyr or Cys (34), making it difficult to type HLA accurately by less sensitive algorithms. Researchers have also demonstrated that HLA-A*02:07 is the most common HLA-A2 subtype among Chinese (35), and the HLA-A*02:07 peptide binding repertoire is limited to a subset of the HLA-A*02:01 repertoire (36), so we need to pay more attention to this allele in practice when these algorithms are used. Except for HLA-A*02:07 allele, HLA-A*11:01 allele had the second highest frequency of miscall for HLA-A gene family. We found that HLAforest was more prone to miscall HLA-A*02:07 allele, while HLAminer had a higher miscall frequency for HLA-A*11:01 in our benchmarked samples. As for HLA-B gene, HLA-B*13:01 is the most frequently miscalled alleles by HLA-VBSeq and HLAforest, while HLA-B*58:01 is inclined to be miscalled by HLAminer and Seq2HLA. As for HLA-C gene, HLA-C*03:02 and HLA-C*03:03 is inclined to be miscalled by HLAminer and Seq2HLA, while HLA-C*01:02 are more frequently miscalled by HLAforest and HLA-VBSeq (the top two miscall patterns for each gene are summarized in **Supplementary Table 3**). These miscall patterns demonstrated that each algorithm had specific systematical bias, which need to be taken into account when developing more accurate algorithm in future.

One of the drawbacks of this study was that only seven HLA typing algorithms (which were selected considering the ease of use of the software and the number of citations of the corresponding articles) were used in this benchmarking evaluation. For example, Polysolver (37) is not evaluated in this study because it depend on Novoalign, which requires commercial components and is also not executable for us because of the incompatible Linux version. Besides, it is reported that the concordance of HLA typing by the current gold standard methods (PCR-based) is only 84%, reflecting the inaccuracy of the laboratory methods as well as inter-laboratory variability (26). We used NGSgo-AmpX as our benchmarked assay, which is a Research Use Only (RUO) and the only one CE-marked IVD product when our study started, and yielded almost 100% homology results compared to Sanger sequencing (38). In addition, seq2HLA and HLAforest are originally used for RNA-seq based HLA typing, they perform best on RNAseq data as the datatype they were designed for, whereas the performance in WES/WGS data decreased significantly (26). Finally, all algorithms were run with their default parameters or the default script without any modification, which may not represent the best performance of the algorithms and could affect the accuracy of HLA typing, it may be the case for HLA-VBseq. There is one more thing that should not be overlooked, the default HLA reference sequence file used in our analysis was provided by the software itself and may be derived from different version of IPD-IMGT/HLA reference database, which may compromise the performance of these software. According to the recommendations of American Society for Histocompatibility and Immunogenetics (ASHI) in regards to NGS based HLA typing, the database which is used for HLA typing should be updated at least every 12 months with the most recent version of the IPD-IMGT/HLA database, and for accredited laboratories, documents should record which version of the IPD-IMGT/HLA or other appropriate nucleotide sequence database was used for allele interpretation. Thus, beside HLA typing accuracy and resolution, selecting a suitable software that have good version control and could regularly update the HLA reference database is of great importance and should never be ignored in clinic.

## Data Availability Statement

The datasets presented in this article are not readily available because human genetic resource data should not be publicly available due to the Regulations on Management of Human Genetic Resources (Guo Ling No.717). Requests to access the datasets should be directed to HC, hao.chen1@3dmedcare.com.

## Ethics Statement

The studies involving human participants were reviewed and approved by the Research Ethics Committee of the First Affiliated Hospital, College of Medicine, Zhejiang University (2017KYKSD678-1). The patients/participants provided their written informed consent to participate in this study.

## Author Contributions

PL, AZ, YG, YS, and HC conceived this study. PL, AZ, YG, RM, and MY provided samples. YS and YC performed the bioinformatics analyses. PL, YS, HD, FL, YY, XL, and HC wrote the manuscript. AZ provide valuable suggestions and comments during the interactive review. All authors contributed to the article and approved the submitted version.

## Funding

This work was funded by the National Key Research and Development Program of China (2017YFC0908500).

## Conflict of Interest

All authors affiliated with 3D Medicines Inc. are current or former employees. There are no patents, products in development or marketed products to declare. The authors declare that the research was conducted in the absence of any commercial or financial relationships that could be construed as a potential conflict of interest.
